# *KCNA2*-Related Epileptic Encephalopathy

**DOI:** 10.15844/pedneurbriefs-29-4-2

**Published:** 2015-04

**Authors:** Jennifer A. Kearney

**Affiliations:** 1 Department of Pharmacology, Northwestern University Feinberg School of Medicine, Chicago, IL

**Keywords:** KCNA2, Dominant-Negative Effect, Epilepsy

## Abstract

Investigators from the University Leipzig and University of Tübingen report mutations of *KCNA2* as a novel cause of epileptic encephalopathy.

Investigators from the University Leipzig and University of Tübingen report mutations of *KCNA2* as a novel cause of epileptic encephalopathy. They identified four de novo mutations in *KCNA2* in six unrelated patients using next generation sequencing.

Four individuals had *KCNA2* mutations that resulted in dominant-negative loss of protein function. Seizure onset was at 8-17 months. EEG became abnormal with multifocal epileptiform discharges. All had delayed speech development and mild to moderate intellectual disability. All became seizure free between 4-15 years of age. Two additional individuals presented with a more severe epileptic encephalopathy phenotype. They had an earlier age of onset, pharmacoresistant seizures, generalized epileptiform discharges with background slowing on EEG, moderate to severe intellectual disability, and moderate to severe ataxia. *KCNA2* mutations associated with the more severe phenotype resulted in pronounced gain-of-function effects. [1]

COMMENTARY. *KCNA2* joins a growing list of voltage-gated potassium channel genes associated with epileptic encephalopathy, including *KCNQ2*, *KCNQ3*, *KCNT1* and *KCNB1* [1–4]. *KCNA2* encodes K_V_1.2, a voltage-gated potassium channel subunit that contributes to repolarization of the neuronal membrane following an action potential. Mutations in *KCNA2* that interfere with normal Kv1.2 function result in impaired electrical signaling and altered membrane excitability. Loss-of-function mutations are predicted to impair membrane repolarization, resulting in neuronal hyperexcitability and a propensity for repetitive firing. Consistent with this, complete absence of K_V_1.2 in homozygous mice resulted in spontaneous seizures and premature death, and heterozygous deletion resulted in increased seizure susceptibility [5]. Mutations were also identified that exhibited gain-of-function effects [1]. At the level of a single neuron, the observed effects predict K_V_1.2 channels that are open at resting membrane potentials, resulting in neuronal hypoexcitability. However, based on the more severe phenotype of the patients, the net effect within neuronal networks is hyperexcitability. Additional studies will be required to determine the effect of *KCNA2* mutations at the level of the network.

Thus far, the phenotypes associated with *KCNA2* mutations comprise two distinct groups based on age of onset, seizure semiology, and electroclinical features. These distinctive clinical phenotypes appear to correlate with differential effects of the mutations on protein function [1]. Patients with gain-of-function mutations have a more severe phenotype and do not achieve seizure freedom. In contrast, patients with loss-of-function mutations have later seizure onset, and achieve seizure freedom in childhood. This nascent genotype-phenotype correlation is reminiscent of *KCNQ2*-associated epileptic encephalopathy, where loss-of-function mutations are associated with neonatal onset, while gain-of-function mutations are associated with infantile onset [3, 6]. Studies with *KCNA2* mutations from additional patients will be required to confirm the genotype-phenotype relationship. If the relationship holds, it will be useful for predicting disease progression and guiding management.
